# Assessing the accuracy of a new hand hygiene monitoring device (SmartRub®): from the laboratory to clinical practice

**DOI:** 10.1186/s13756-021-01026-2

**Published:** 2021-11-06

**Authors:** Chloé Guitart, Yves-Alain Robert, Nasim Lotfinejad, Simon Fourquier, Yves Martin, Daniela Pires, Julien Sauser, René Beuchat, Didier Pittet

**Affiliations:** 1 Infection Control Programme and WHO Collaborating Centre On Patient Safety - Infection Control and Improving Practices, University of Geneva Hospitals and Faculty of Medicine, Geneva, Switzerland; 2 iQati, Sion, Switzerland; 3 Haute école du paysage, d’ingénierie et d’architecture de Genève (HEPIA), Geneva, Switzerland

**Keywords:** Hand hygiene, Hospital acquired infections, Electronic monitoring device, Sensitivity, Specificity

## Abstract

**Background:**

We developed SmartRub® powered by iQati®, an electronic device composed of a wristband and an alcohol-based handrub pocket-sized dispenser that measures and provides feedback on the duration of hand friction and the volume poured during each hand hygiene action. We aimed to assess the accuracy of SmartRub®.

**Methods:**

The specificity, sensitivity, positive and negative predictive values (PPV and NPV) of SmartRub® were assessed in a 3-phased experiment: (1) laboratory-controlled conditions with volunteers; (2) pre-planned clinical path with volunteers and (3) real clinical conditions with healthcare workers. The accuracy of SmartRub® was evaluated by quantifying its ability to correctly capture true hand hygiene actions and to not record other actions performed while wearing the device.

**Results:**

In the laboratory, 7 volunteers performed 816 actions. Overall sensitivity was 94.1% (95% CI 91.4–96.2%) with a PPV of 99.0% (95% CI 97.3–99.6%) and specificity was 99.0% (95% CI 97.5–99.7%) with a NPV of 94.4% (95% CI 91.9–96.1%). During the pre-planned clinical path phase, 13 volunteers performed 98 planned paths and a total of 967 actions were performed. Overall sensitivity was 94.6% (95% CI 92.2–96.5%) with a PPV of 84.3% (95% CI 81.6–86.7%) and specificity was 82.4% (95% CI 78.7–85.7%) with a NPV of 93.9% (95% CI 91.3–95.7%). During the real clinical conditions phase, 17 healthcare workers were observed for a total of 15 h and 3 min while they performed 485 actions. Sensitivity was 96.8% (95% CI 93.8–98.6%) with a PPV of 98.3% (95% CI 95.6–99.3%) and specificity was 98.3% (95% CI 95.7–99.5%) with a NPV of 96.8% (95% CI 93.9–98.4%).

**Conclusions:**

Smartrub® is a highly reliable device for capturing hand hygiene actions under a range of conditions, from the laboratory to clinical care activities.

## Background

Healthcare-associated infections (HAIs) are the most frequent adverse events occurring during patient care. According to the World Health Organization (WHO), 7% of patients acquire at least one HAI during hospitalization in developed countries and HAI rates may be as high as 17% in developing countries [1, 2].

Compelling evidence shows that inadequate hand hygiene (HH) performance leads to cross-transmission of microorganisms and HAI [3, 4]. The WHO HH guidelines state that performing the recommended HH actions at the right moments is the most effective way to prevent HAI [3]. However, HH compliance remains low in healthcare worldwide [5]. The WHO multimodal strategy for HH improvement states that monitoring and feedback of HH practices are essential to achieve best practices [6]. To complement the WHO method of direct observation, some healthcare facilities are using additional interventions such as electronic dispensing counters and automated HH monitoring networks (including wearable devices) [7]. These automated systems likely require fewer human resources, provide larger and more representative data sets, and are less subject to observation bias than the WHO recommended direct observation method [8–10]. However, fewer than 20% of the HH monitoring systems included in published studies were subject to accuracy testing [7]. Importantly, Healthcare workers (HCWs) frequently identify lack of system accuracy as undermining their trust in the tool [11].

SmartRub® powered by iQati® (SmartRub®) is a new electronic wearable device that monitors the quality of individual HCW’s HH actions and provides HCWs with feedback. SmartRub® was developed by a partnership between the University of Geneva Hospitals (HUG) and Faculty of Medicine, the “Haute école du paysage, d’ingénierie et d’architecture de Genève” (HEPIA) and iQatiTM, a start-up company. We aimed to test the accuracy of SmartRub® during three increasingly challenging scenarios, from laboratory controlled conditions to daily clinical activities at the University Hospitals of Geneva.

## Methods

### Setting

The study was conducted at the University Hospitals of Geneva (HUG), a 1,900-bed tertiary-care university hospital center with 60,000 hospital admissions per year and covering a population of about 800,000 inhabitants.

### Hand hygiene monitoring device: SmartRub®

**Fig. 1:**
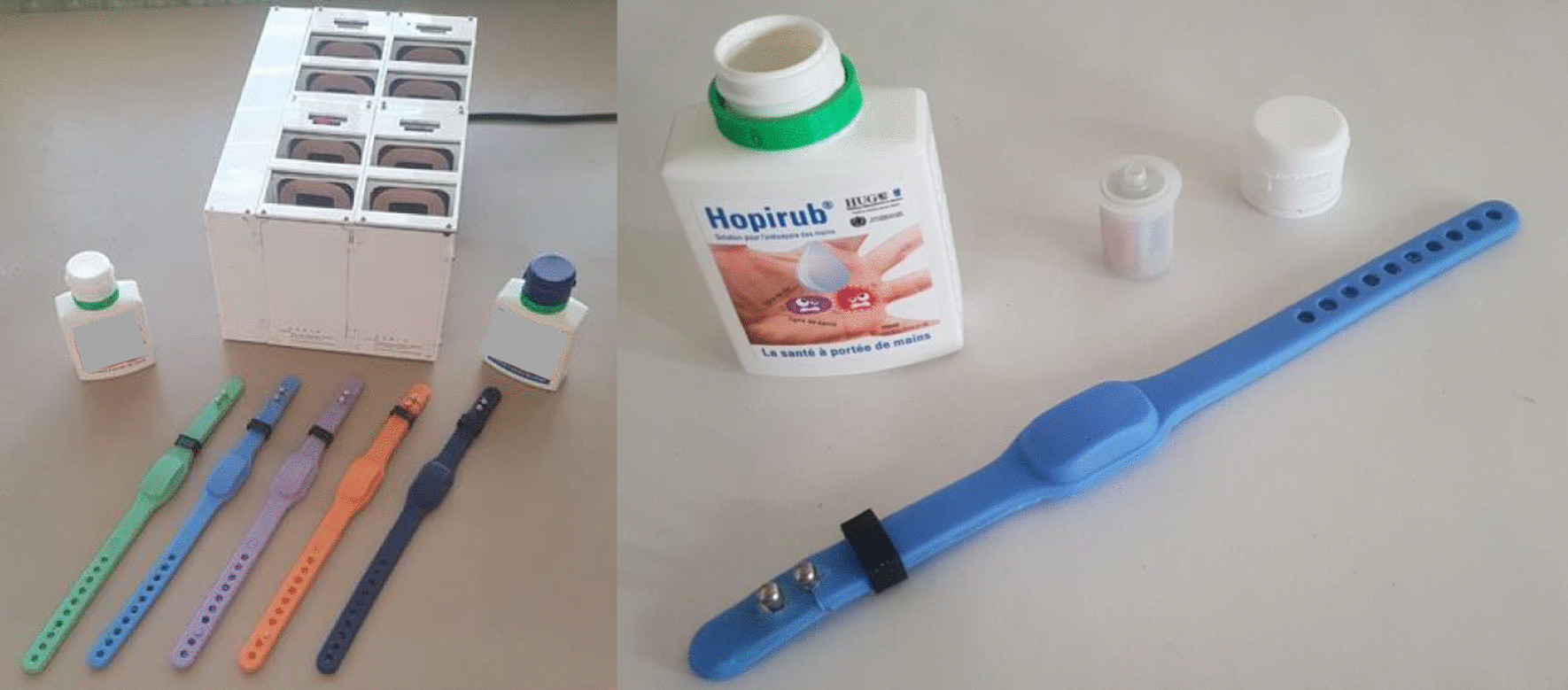
Three hardware components of SmartRub® device: the station, the bracelet and the cylinder

Smartrub® has four main components (Fig. 1): (1) a cylinder adapted to the 100 mL individual alcohol-based handrub (ABHR) pocket bottle used at HUG, containing a turbine, a vibrator and a battery that measure the volume dispensed each time the bottle is used; (2) a wristband made of medical silicone, housing an accelerometer, a vibrator and a battery that are activated by the use of the bottle and then measure the duration of hand friction and give the HCW feedback; (3) a recharging station where the bottle and the wristband are placed when not in use; and (4) a secure server that receives the data from the cylinder and wristband when they are in the recharging station. In addition to monitoring both the volume of ABHR used (cylinder) and the duration of handrubbing (wristband) for each HH action performed by the HCW, SmartRub® also vibrates to provide immediate and personalized positive feedback to the HCW on these two parameters.

The amount of ABHR each HCW should dispense was adjusted to the HCWs’ hand size and the feedback of the bracelet was set to be received after 15 s of handrubbing, based on results of previous laboratory studies [12, 13]. The error on the volume measurement averaged − 0.02 ± (SD) 0.1 mL and the error on the duration − 0.1 ± (SD) 1.4 s (unpublished data).

### Validation approach

We adapted the 3-phase validation approach described by Limper et al. [14] to assess the specificity, the sensitivity, the positive predictive value (PPV) and the negative predictive value (NPV) of SmartRub®. During phase I, we evaluated the accuracy of Smartrub® under controlled laboratory conditions with trained volunteers who performed pre-determined actions. In the second phase, trained volunteers followed a pre-planned sequence of care in an empty hospital ward, mimicking a defined clinical care situation. Phase III tests were performed by HCWs during their daily clinical activities and their actions were noted by trained observers.

**Fig. 2:**
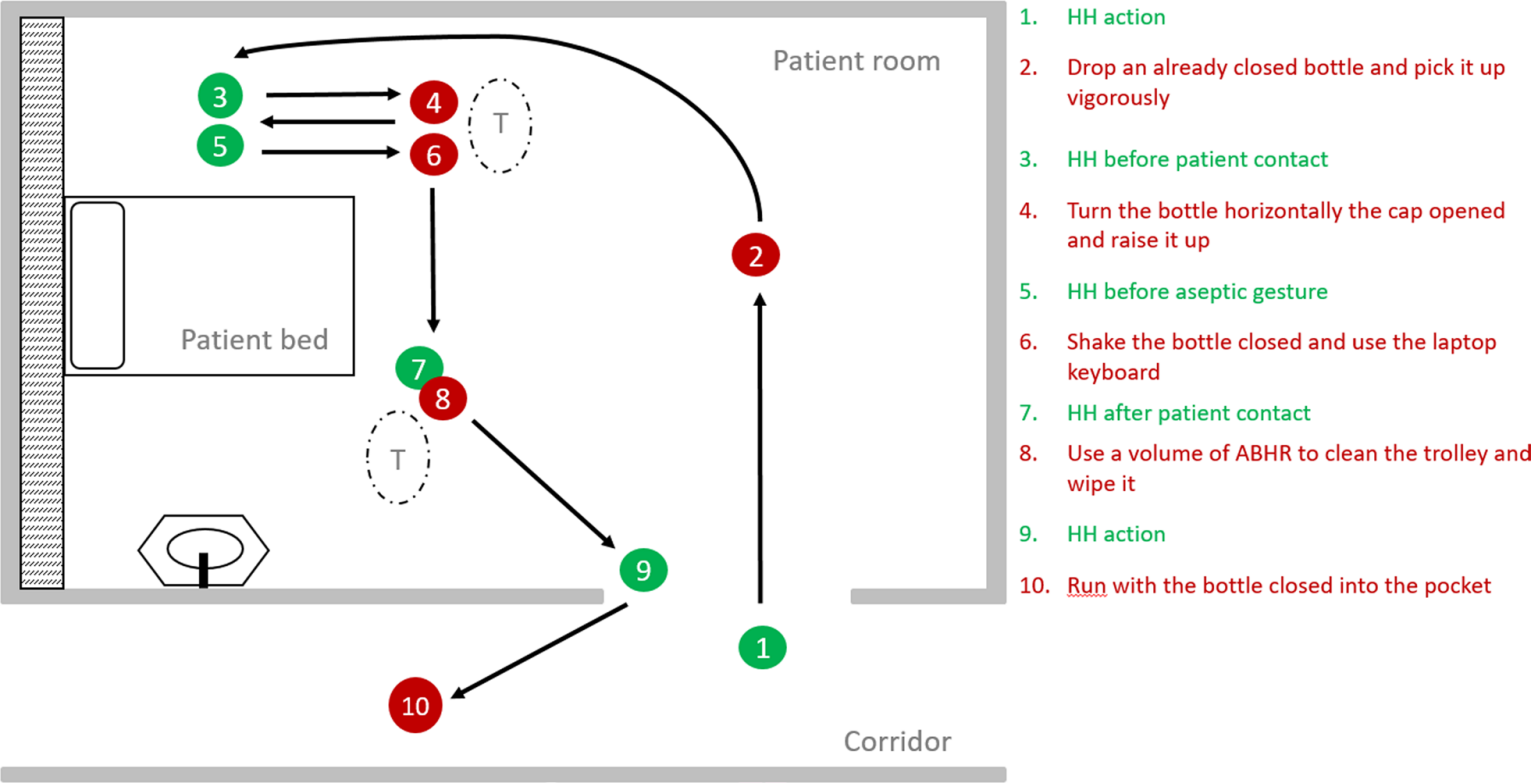
Pre-planned path of five true actions and five false actions

During phases I and II, we defined true actions (HH related actions) and false actions (other specific actions performed by participants while wearing the device). During all phases, an observer noted when the HH actions were performed. We compared these actions with the data captured by the device. The study team defined all actions and the pre-planned clinical path during consensus building discussions (Fig. 2). During all phases, the ABHR tested was the standard product used at HUG (100 mL, containing isopropyl alcohol 68.5%, chlorhexidine digluconate 0.58% and propan-2-ol, Hopirub® or Hopigel®, BBraun, Switzerland).

### Phase I: controlled laboratory conditions

During phase I, we first assessed the ABHR bottle and then the overall device (wristband + bottle) combined for each hand rub formulation (gel and rinse). The wristband was not assessed independently because it starts measuring the duration of hand rubbing only after the bottle activation. Volunteers were given the list of alternate true and false actions to be performed every minute and were supervised by a trained observer to ensure correct compliance. True actions with the bottle consisted of pouring different volumes controlled into a graduated cylinder. Wristband true actions were the duration of hand friction measured by a timer and different speeds according to volunteer preference. Alternatively, we performed 6 false actions and 3 true actions described in Table 1 in order to challenge the device (Table 1). For tests of both the bottle and the wristband, we assessed a combination of true or false actions with the bottle and wristband sequentially.

**Table 1:** Phase 1 (laboratory conditions) predetermined true and false actions of the bottle and the wristband

Bottle actions	Wristband actions
*True actions*	
Pouring a volume < 1.5 mL	Slow HH action during [0; 10 s]; [10–15 s] or [15; ∞[
Pouring a volume [ 1.5–2.5 mL]	Fast HH action during [0; 10 s]; [10–15 s] or [15; ∞[
Pouring a volume > 2.5 mL	Usual HH action during [0; 10 s]; [10–15 s] or [15; ∞[
*False actions*	
Displacing horizontally the bottle with cap closed	Hands into the pockets
Displacing horizontally the bottle with cap open	Moving the bottle with the cap closed
Bottle falling down the floor with cap closed	Cleaning the table
Bottle falling down the floor with cap open	Start writing
Shaking the bottle with cap closed Shaking the bottle with cap open	(all actions were performed after an action of the bottle)

### Phase II: pre-planned clinical path

During the second phase, trained volunteers performed the pre-planned path in an empty hospital ward at HUG. The pre-planned path consisted of five true actions (real HH action gestures) alternated with five false actions (actions resembling but not related to HH), precisely defined in time and space along the planned path (Fig. 2). To assess if the feedback influenced the device accuracy, we performed half of the study with and half without feedback activation.

### Phase III: clinical care conditions

The third and last phase was performed under real life clinical conditions. HCWs from the ambulatory surgical ward at HUG volunteered to wear SmartRub® devices during their daily patient care activities. Observers documented all consecutive HH actions performed by volunteers by direct observation. Data were collected via a smartphone application and included the exact time of each HH action and the device code tested by each volunteer. A false action was defined as all gestures performed by HCWs between two true HH actions. During this phase, the feedback was not activated and volunteers used their preferred ABHR formulation (gel or rinse).

#### Sample size calculation

We adapted sample size calculations from the literature regarding validation of new diagnostic and screening tests [15]. We set the prevalence of HH behaviors at 50%. Overall, HH compliance at HUG is 70% on average since 2011, but we defined HH behavior prevalence for the sample size calculation as an estimate of the proportion of all HCW movements on the hospital ward that was related to HH (e.g., before and after touching a patient, before performing an aseptic task, after contact with body fluid and after touching the patient’s environment). We based this conservative estimate on our routine observations of HCWs’ practices. We set the power to be at least 80% and the type I error at 0.05 considering a two-sided test. Based on unpublished pilot data, we set the null hypothesis for sensitivity and specificity for the phase I experiment with the bottle only at 90% and the alternative hypothesis at 95%. For the phase I combined experiment (bottle and wristband), we set the null hypothesis for a sensitivity and a specificity at 50% and the alternative hypothesis at 60%. A sample size of 462 actions for the bottle only experiment and 398 actions for the device combined, of which half were true actions and half were false actions, was necessary to accurately estimate the sensitivity, specificity, PPV and NPV of the device. Based on data from the first phase, we set the sensitivity and the specificity, at 90% (null hypothesis) and the alternative hypothesis at 95% for the phase 2 and phase 3 experiments, and we set the sample size at 462 actions for each phase, of which half were true actions and half were false actions.

#### Statistical analysis

The true actions/false actions performed by participants and observed by trained volunteers were considered the gold standard. We then compared this information with the data captured by the device. We treated each device encounter as an independent event, even though each volunteer performed several measures. If the device correctly recorded a true action (with 5 s more or less time limit, corresponding to the observer's reaction time), we considered it to be a true positive. If the device recorded a false action, we considered it to be a false positive. Otherwise, if the device did not record a false action, we would classify it as true negative and if the device did not record a true action, this would be classified as a false negative. During phase III, we considered a false positive as any action recorded by the device in the time period between two HH actions and a true negative if there was no record in this time lapse. During all phases, any action recorded by the device that did not correspond to a true action would be considered a false positive.

We defined sensitivity as the probability that a true action was captured by the system, specificity as the probability that a false action was captured by the system, the PPV as the probability that the activity captured by the system really occurred, and the NPV as the probability that no HH action occurred when no activity was captured by the system. For all phases, we calculated point estimates of sensitivity, specificity, PPV and NPV and the 95% confidence intervals (95% CI) using the methodology described by Mercaldo et al. [16]. We used R (version 3.6.3) for the analyses.

## Results

Overall, 1382 true actions and 1384 false actions were performed in phase I and II. In phase III, 249 true actions were performed (Table 2). The sensitivity, specificity, PPV and NPV (as well as 95% CI around the estimates) of SmartRub® device in phase I, II and III are depicted in Table 3. The sensitivity and NPV did not differ significantly among all phases. In contrast, the specificity and PPV were significantly lower in the pre-planned clinical path compared with that for device testing in laboratory conditions and the clinical activities.

**Table 2:** Numbers of actions performed in each study phase

	True positive (n)	False negative ( n)	True negative (n)	False positive (n)
*Phase 1 (bottle only)*				
Gel	214	22	238	0
Rinse	252	2	232	23
Total	466	24	470	23
*Phase 1 (bottle and wristband)*				
Gel	188	16	204	0
Rinse	196	8	200	4
Total	384	24	404	4
*Phase 2*				
Feedback off	223	20	201	43
Feedback on	235	6	197	42
Total	458	26	398	85
*Phase 3*				
Total	241	8	231	5

Phase 1, controlled laboratory condition; Phase 2, pre-planned clinical path; Phase 3, clinical care condition; N, number of actions

**Table 3:** Sensitivity, specificity, PPV and NPV of SmartRub® device in phase I (part 1 and 2), II and III

	Sensitivity % (95% CI)	Specificity % (95% CI)	PPV % (95% CI)	NPV % (95% CI)
*Phase 1 (bottle only)*				
Gel	90.7 (86.2–94.1)	100.0 (98.5–100.0)	99.4 (96.5–99.8)	91.0 (87.2–93.6)
Rinse	99.2 (97.2–99.9)	91.0 (86.8–94.2)	91.7 (88.2–94.2)	99.1 (96.7–99.8)
*Phase 1 (bottle and wristband)*				
Gel	92.2 (87.6–95.5)	100.0 (98.2–100.0)	99.3 (96.0–99.8)	92.2 (88.1–94.7)
Rinse	96.1 (92.4–98.3)	98.0 (95.1–99.5)	98.0 (94.9–99.2)	96.2 (92.7–98.0)
*Phase 2*				
Gel	91.8 (87.6–94.9)	83.5 (78.3–88.0)	84.8 (80.7–88.1)	91.0 (86.9–93.9)
Rinse	97.5 (94.7–99.1)	81.2 (75.7–86.0)	83.9 (80.0–87.1)	97.0 (93.7–98.6)
Feedback off	91.8 (87.6–94.9)	82.4 (77.0–86.9)	83.9 (79.8–87.3)	90.9 (86.8–93.9)
Feedback on	97.5 (94.7–99.1)	82.4 (77.0–87.0)	84.7 (80.8–88.0)	97.1 (93.7–98.7)
*Phase 3*				
Total	96.8 (93.8–98.6)	98.3 (95.7–99.5)	98.3 (95.6–99.3)	96.8 (93.9–98.4)

95% CI, confident interval 95%; PPV, positive predictive value; NPV, negative predictive value; Phase 1, controlled laboratory condition; Part 1, bottle only, Part 2, bottle and wristband combined; Phase 2, pre-planned clinical path; Phase 3, clinical care condition

In the first part of the phase I experiment, 3 volunteers tested 6 bottles of ABHR by performing 983 actions, 490 of which were true and 493 false. Of these, 510 actions were performed with ABHR rinse and 476 with ABHR gel. The use of rinse was associated with a significantly higher sensitivity and a significantly lower specificity compared to the gel formulation. Overall, the sensitivity was 95.1% (95% CI 92.8–96.8%), specificity 95.3% (95% CI 93.1–97.9%), PPV 95.3% (95% CI 93.2–96.8%) and NPV 95.1% (95% CI 92.9–96.6%) in this phase. During phase I, part 2 assessing both the bottle and the wristband combined, 7 volunteers performed 816 actions, half of which were true and half false. They performed 408 actions with ABHR rinse and 408 with ABHR gel. The device sensitivity, specificity, PPV and NPV did not differ significantly between the rinse and gel formulation. Overall, the sensitivity was 94.1% (95% CI 91.4–96.2%), specificity 99.0% (95% CI 97.5–99.7%), PPV 99.0% (95% CI 97.3–99.6%) and NPV 94.4% (95% CI 91.9–96.1%) in this phase.

During phase II, 13 volunteers performed 98 planned paths and 967 actions, of which 484 were true actions and 483 were false actions. In total, 10 ABHR dispensers (5 gel; 5 rinse) and 10 bracelets were used. A total of 480 actions were performed with the feedback activated and 487 without. The sensitivity, specificity, PPV and NPV did not significantly differ when the feedback was activated. A total of 481 actions were performed using the rinse and 486 using the gel. The device sensitivity, specificity, PPV and NPV did not differ significantly between rinse and gel formulation. Overall, the sensitivity was 94.5% (95% CI 92.2–96.5%), specificity 82.4% (95% CI 78.7–85.7%), PPV 84.3% (95% CI 81.6–86.7%) and NPV 93.9% (95% CI 91.3–95.7%) in this phase.

During phase III, 17 volunteers participated and were observed for a total of 15 h and 3 min. They performed 485 actions, of which 249 were true actions and 236 were false actions. In total, 92% of true actions were performed using an ABHR rinse and only 8% using the gel formulation. Overall, the sensitivity was 96.8% (95% CI 93.8–98.6%), specificity 98.3% (95% CI 95.7–99.5%), PPV 98.3% (95% CI 95.6–99.3%) and NPV 96.8% (95% CI 93.9–98.4%) in this phase.

## Discussion

The overall sensitivity and NPV of the device were high and similar in the three phases of the study. In contrast, the specificity and PPV did not differ between phase I and phase III but were significantly lower during phase II that in phase I or phase III. We think the action “cleaning the table” increased the number of false positive measures in the pre-planned clinical path phase and HCWs likely did not perform this action under real clinical conditions. However, the fact that they did not perform this action could be due to the presence of observers. During phase I, when only the bottle of ABHR was tested, the sensitivity and NPV were significantly higher for the rinse formulation compared with the gel formulation, and inversely, the specificity and PPV were higher for the gel formulation than for the rinse formulation. The captor in the bottle cap is a turbine and the rinse formulation passes easily through the turbine improving the sensitivity of the system. The difference between the formulations was not evident during phase I, part 2 and during phase II suggesting that the connection with the bracelet may mitigate the difference in accuracy between the rinse and gel formulations. During phase II, the sensitivity, specificity, PPV and NPV did not change significantly when the feedback was activated, but the study was not powered to detect such a difference.

The SmartRub® device is unique, to the best of our knowledge, and the first device capable of measuring the volume of ABHR used and the duration of hand friction. Other electronic HH monitoring devices described in the literature include network monitoring systems capturing room and/or patient zone entries and exits and HCWs’ HH events by radiofrequency, infrared thermal or wireless identification-based systems. Only a few studies tested the accuracy of the device using a methodology similar to our simulated conditions and real clinical conditions. One study found a sensitivity of 88.7% under simulated conditions and 92.7% under real clinical conditions [14]. Another study found that the accuracy of measuring HH events decreased from regarding 88.5% under simulated conditions to 52.4% under real clinical conditions [17]. We cannot directly compare SmartRub’s® accuracy in detecting HH actions with that of the other devices. However, our data suggest that SmartRub® may be more accurate compared to the other electronic HH monitoring systems in the literature. Our objective measures of sensitivity and specificity indicate that SmartRub® may enable us to capture HCWs’ behaviors associated with ABHR under clinical conditions.

We conducted the current study to assess of the accuracy of the SmartRub® device in capturing HH actions before testing its capacity to improve the quality of HH gestures among HCWs. A recent systematic review on the effectiveness of automated HH monitoring systems in health care settings found that fewer than 20% of the articles assessed the systems’ accuracy or predictive values [7]. Given that the volume of ABHR dispensed and the duration of HH are major determinants of HH’s antimicrobial efficacy [12, 18], we suggest that the accuracy of a HH monitoring device must be assessed before its effectiveness and before it is marketed.

One of the limitations of this study that was observed during phase III is that the device accuracy was compared to human evaluation of hand hygiene performance, which depends on the expertise of the HCW in hand hygiene observation. In order to overcome this limitation, we recruited validated observers in our study. However, the accuracy of SmartRub® should be further studied in other settings, as the findings of this study may not be generalizable based on the results of only one ward. In addition, due to the presence of the observers, observation bias could also be mentioned when evaluating the performance of other HCWs.

We observed that the SmartRub® device is highly accurate when tested with the triple-validation approach. Our results demonstrate the capacity of this device to capture HH actions with both high sensitivity and high specificity. This sensitivity and specificity assessment was an important step between our engineering validation and our planned clinical effectiveness evaluation. The results of this study suggest that the SmartRub® device is a promising tool for enhancing HCWs’ HH quality, thus supporting a key element in infection prevention and control.

## Data Availability

The datasets generated during and/or analysed during the current study are available by email.

## References

[1] Allegranzi B, Bagheri Nejad S, Combescure C, Graafmans W, Attar H, Donaldson L (2011). Burden of endemic health-care-associated infection in developing countries: Systematic review and meta-analysis. Lancet Lond Engl..

[2] Point prevalence survey of healthcare-associated infections and antimicrobial use in European acute care hospitals 2011–2012. European Centre for Disease Prevention and Control. 2013 [cité 15 avr 2020]. Disponible sur: https://www.ecdc.europa.eu/en/publications-data/point-prevalence-survey-healthcareassociated-infections-and-antimicrobial-use-0.

[3] World Health Organization, WHO Patient Safety. WHO guidelines on hand hygiene in health care : a summary. Résumé des recommandations de l’OMS pour l’hygiène des mains au cours des soins. 2009 [cité 15 avr 2020]; Disponible sur: https://apps.who.int/iris/handle/10665/70126.

[4] Laustsen S, Lund E, Bibby BM, Kristensen B, Thulstrup AM, Kjølseth MJ (2008). Effect of correctly using alcohol-based hand rub in a clinical setting. Infect Control Hosp Epidemiol.

[5] Pittet D (2008). Hand hygiene: it’s all about when and how. Infect Control Hosp Epidemiol.

[6] World Health Organization, WHO Patient Safety. A guide to the implementation of the WHO multimodal hand hygiene improvement strategy. 2009 [cité 15 avr 2020]; Disponible sur: https://apps.who.int/iris/handle/10665/70030.

[7] Ward MA, Schweizer ML, Polgreen PM, Gupta K, Reisinger HS, Perencevich EN (2014). Automated and electronically assisted hand hygiene monitoring systems: a systematic review. Am J Infect Control mai.

[8] Dhar S, Tansek R, Toftey EA, Dziekan BA, Chevalier TC, Bohlinger CG (2010). Observer bias in hand hygiene compliance reporting. Infect Control Hosp Epidemiol août.

[9] Boyce JM (2017). Electronic monitoring in combination with direct observation as a means to significantly improve hand hygiene compliance. Am J Infect Control.

[10] Pires D, Pittet D (2017). Hand hygiene electronic monitoring: Are we there yet?. Am J Infect Control.

[11] Dyson J, Madeo M (2017). Investigating the use of an electronic hand hygiene monitoring and prompt device: influence and acceptability. J Infect Prev.

[12] Bellissimo-Rodrigues F, Soule H, Gayet-Ageron A, Martin Y, Pittet D (2016). Should alcohol-based handrub use be customized to healthcare workers’ hand size?. Infect Control Hosp Epidemiol févr.

[13] Paula H, Becker R, Assadian O, Heidecke C-D, Kramer A (2018). Wettability of hands during 15second and 30-second handrub time intervals: A prospective, randomized crossover study. Am J Infect Control.

[14] Limper HM, Slawsky L, Garcia-Houchins S, Mehta S, Hershow RC, Landon E (2017). Assessment of an aggregate-level hand hygiene monitoring technology for measuring hand hygiene performance among healthcare personnel. Infect Control Hosp Epidemiol.

[15] Bujang MA, Adnan TH (2016). Requirements for minimum sample size for sensitivity and specificity analysis. J Clin Diagn Res.

[16] Confidence intervals for predictive values with an emphasis to case–control studies—Mercaldo—2007—Statistics in Medicine—Wiley Online Library. [cité 17 juill 2020]. Disponible sur: 10.1002/sim.2677 10.1002/sim.2677 16927452

[17] Pineles LL, Morgan DJ, Limper HM, Weber SG, Thom KA, Perencevich EN (2014). Accuracy of a radiofrequency identification (RFID) badge system to monitor hand hygiene behavior during routine clinical activities. Am J Infect Control févr.

[18] Pires D, Soule H, Bellissimo-Rodrigues F, Gayet-Ageron A, Pittet D (2017). Hand hygiene with alcohol-based hand rub: how long is long enough?. Infect Control Hosp Epidemiol.

